# Assessment of Welfare Problems in Finnish Cattle and Pig Farms Based on Official Inspection Reports

**DOI:** 10.3390/ani9050263

**Published:** 2019-05-22

**Authors:** Sofia Väärikkälä, Laura Hänninen, Mari Nevas

**Affiliations:** 1 Department of Food Hygiene and Environmental Health, Faculty of Veterinary Medicine, University of Helsinki, P.O. Box 66 (Agnes Sjöbergin katu 2), 00014 Helsingin Yliopisto, Finland; mari.nevas@helsinki.fi; 2 Research Centre for Animal Welfare, Faculty of Veterinary Medicine, University of Helsinki, P.O. Box 57, 00014 Helsingin Yliopisto, Finland; laura.hanninen@helsinki.fi; 3 Department of Production Animal Medicine, Faculty of Veterinary Medicine, University of Helsinki, P.O. Box 57 (Viikintie 49), 00014 Helsingin Yliopisto, Finland

**Keywords:** animal welfare, cattle farms, inspections, non-compliance, pig farms

## Abstract

**Simple Summary:**

Official on-farm inspections are carried out throughout the European Union every year to ensure farm compliance with animal welfare standards. The aim of this study was to analyze Finnish inspection data in order to find out how well cattle and pig farms comply with animal welfare standards, to reveal the most common non-compliances and to identify possible farm risk factors. About every fourth inspected Finnish cattle and pig farm did not comply with the animal welfare standards. Examples of factors that increased the risk of non-compliance were small herd size, tie-stall housing and outdoor rearing. Inadequate lying area in cattle farms and a lack of enrichment material in pig farms were the most common non-compliances. The regional differences found may indicate differences in inspectors’ interpretations or ways in conducting inspections. As the official inspection reports contain valuable information about the welfare problems on farms, the reports should be better utilized in risk analysis, in targeting farmer education, and in making the inspections more uniform.

**Abstract:**

The competent authorities of the Member States of the European Union are required to perform animal welfare inspections on livestock farms. The data obtained from these official inspections performed in Finnish cattle and pig farms in 2010–2015 were used with the aim of estimating the prevalence of the most common non-compliances and identifying underlying risk factors. The prevalence of non-compliant cattle and pig farms was 24.2% and 27.9%, respectively. In cattle, the most common problem was an inadequate lying area followed by deficient housing conditions for calves; in pigs, it was a lack of enrichment material. The non-compliances concerning cattle were most frequently detected in autumn and in farms with small herd size, with tie-stall housing and outdoor rearing year-round. The pig farms with a farrow-to-finish unit had a higher prevalence of non-compliances than other production types. The prevalence of the non-compliant farms differed notably between the regions. It can be concluded that the cattle welfare inspections should be performed with a focus on the cold and rainy seasons and at small farms, whereas the pig welfare inspections should mainly focus on farrow-to-finish units. The data received from official inspections should be efficiently utilized in the development of animal welfare inspection system, with the aim of risk-based, consistent and uniform inspections. In addition, the data should be utilized in targeting information for farmers.

## 1. Introduction

A framework for the protection of farmed animals in the European Union (EU) is provided by the current animal welfare standards, which are based on Council Directive 98/58/EC concerning farmed animal welfare [1] and on the directives adopted pursuant to it. These rules lay down minimum standards for the physical environment and resources available to the animal, such as freedom of movement, housing, feeding and watering, and the management of animals, such as how often animals are inspected or fed. According to the European Food Safety Authority, an assessment verifying compliance with legislative standards usually focuses on factors that can prevent poor animal welfare, e.g., floor type or provision of water [2]. The need for the assessment of these factors in highlighting the potential risk of reduced welfare and identifying the underlying reasons for current animal welfare problems has been previously recognized [3].

EC Regulation No 882/2004 [4] requires EU Member States to ensure that on-farm inspections are carried out by competent authorities to evaluate compliance with animal welfare standards. There are no guidelines for the minimum number of inspections required per year, but the sample size must be comprehensive. Thus, a considerable amount of data about the factors impacting the welfare of farmed animals is consistently acquired. In Finland, EU animal welfare inspections started in 1998 with calf and pig farms and the inspections have expanded to include fur farms, laying hen, duck, goose and broiler farms as well as sheep, goat and adult cattle farms. Each year approximately 1–7% of Finnish farms to be inspected are chosen both on risk basis (75%) and by random sampling (25%) by the Finnish Food Safety Authority Evira. The examples of risk factors that Evira is taking into consideration when choosing the farms are previously detected welfare problems, previous deficiencies related to marking and registration, and the number of animals [5]. In Finland, the data obtained have not been thoroughly evaluated and utilized for the development of animal welfare inspection system. In Sweden, the inspection data have been used to determine the incidence of specific welfare conditions and risk factors for poor welfare [3,6,7]. In France it has been used to improve compliance with EU animal welfare legislation and the efficiency of the inspection system [8], and in Great Britain to establish whether farm membership of a voluntary assurance, organic or welfare scheme has an impact on compliance with animal welfare legislation [9,10]. The aim of this study was (i) to assess the compliance of the cattle and pig farms of the minimum legal standards for animal welfare and (ii) to identify potential risk factors related to farm type or season. This will help to strengthen the animal welfare inspection system and improve welfare compliance.

## 2. Materials and Methods

Electronic reports of sample-based EU animal welfare inspections carried out by regional authorities on Finnish cattle and pig farms from 1 January 2010 to 31 December 2015 were provided by the National Land Survey of Finland. The data consisted of 1842 inspection reports on cattle farms and 408 reports on pig farms. Of these inspection visits 25% had been performed based on random sampling and 75% on risk basis, based on to a national level evaluation by the Food Safety Authority. Inspections carried out in the Åland Islands (an autonomous region of Finland) and incomplete inspections were excluded. In cases where the farm had been inspected more than once, only the first inspection was included. In all, 1546 inspection reports on cattle farms and 362 reports on pig farms were used in this study.

The inspections and reports are based on detailed checklists covering the standards of national animal welfare legislation. The cattle farm checklist consists of two parts: the first part targeted on adult cattle (cattle over 6 months of age), consisting of 38 requirements (Table 1, first column), and the second on calves, consisting of 39 requirements (Table 2, first column). The pig farm checklist consists of 43 requirements (Table 3, first column). The outcome of each requirement is recorded by the competent authority as compliant, non-compliant or not relevant for the farm in question. The requirements are grouped into six (adult cattle) or five (calves and pigs) main welfare categories: (1) general requirements for premises, (2) space requirements, (3) environment, (4) management, (5) feeding and watering, and (6) outdoor rearing and pasture. The main welfare category is coded as compliant only if all the relevant requirements are complied with. The prevalence of non-compliances was counted from farms in which the requirement was relevant, not from all inspected farms. 

For the data analyses, the data were categorized based on the number of animals, the production type, the farm location and the inspection season. Based on the herd size the farms were divided into small (less than 50 cattle or less than 250 pigs), medium (50–100 cattle or 250–750 pigs) or large (more than 100 cattle or 750 pigs) sized farms. The production types of the cattle farms were dairy cattle, suckler cow herd, or other (including beef production, calf and heifer rearing, and mixed farms). In pig farms, the production types were farrowing, farrow-to-finish, and fattening. The production type was categorized as farrowing if the number of fattening pigs was less than 10% of the number of piglets.

Based on the location, the farms were divided into six regions according to the Regional State Administrative Agencies. As only one pig farm inspection had been performed in Lapland during the study period, Lapland was excluded from the regional analysis concerning pig farms. In other analyses all pig farms were included. In order to discover the relevance of the outdoor temperature and rainfall, these parameters were collected from the archives of the Finnish Meteorological Institute [11]. Furthermore, the inspections were divided into four seasons based on the date of the inspection: winter (December, January, February), spring (March, April, May), summer (June, July, August) and autumn (September, October, November).

The Kolmogorov–Smirnov test was used to determine the distribution of data. As the data were not normally distributed, non-parametric tests were used. Comparisons between different groups were performed with the Mann–Whitney *U* (two groups) or Kruskal–Wallis *H* test (more than two groups). If the Kruskal–Wallis *H* test was statistically significant, the pairwise comparisons were done with the Mann–Whitney *U* test using the Bonferroni correction. The differences between cattle and pig farms were analyzed with Chi square test. Statistical analysis was performed with IBM SPSS Statistics for Windows Version 22.0 (IBM Corp., Armonk, NY, USA). Statistical significance was accepted at a confidence level of 95% (*p* < 0.05).

## 3. Results

### 3.1. Inspected Farms

Based on the cattle farm reports, the inspections concerned adult cattle in 1520 (98.3%) farms and calves in 1345 (87.0%) farms. The division of the inspected farms based on the herd size, the production type, the location and the inspection season is presented in Table 4. The average number of existing Finnish cattle and pig farms during the study period (2010–2015) was 12 869 (range 11,423–14,555) and 1070 (range 787–1355), respectively. These inspected farms approximately constituted 12.0% and 33.8% cattle and pig farms, respectively. The geographical distribution of the inspected farms was congruent with the overall distribution of the farms [12].

### 3.2. Main Non-Compliances

Of all the inspected cattle farms, 24.2% (374/1546, 95% confidence interval (CI) 22.1–26.4) did not comply with the animal welfare standards. This included 18.8% (*n* = 286, 95% CI 16.9–20.8) of the farms with adult cattle and 17.0% (*n* = 229, 95% CI 15.1–19.1) of the farms with calves. An inadequate lying area was the most frequently detected individual non-compliance for the adult cattle, followed by inadequate weather protection, dirtiness of premises and inadequate hoof care (Table 1). For the calves, the most common non-compliances were too small group boxes, overaged calves kept in single pens and an inadequate lying area (Table 2). 

Of the inspected pig farms, 27.9% (*n* = 101, 95% CI 23.5–32.7) did not comply with the animal welfare standards. A lack of enrichment material was clearly the most common non-compliance, followed by insufficient record-keeping of medical treatment and routine grinding or cutting of the piglets’ teeth (Table 3).

The overall non-compliance rates for cattle and pig farms were not statistically significantly different (Chi square test *p* = 0.156), however, the difference was statistically significant for space and management requirements (*p* < 0.001, for both). The non-compliances related to space requirements were more often detected in cattle farms (Table 1) than in pig farms (Table 3) and vice versa for the non-compliances related to management requirements.

### 3.3. Effect of Herd Size

There was a statistically significant difference between the different sized cattle farms in complying with the welfare requirements (Kruskal–Wallis *p* = 0.01). The small cattle farms were more often detected as being non-compliant than the large ones (27.7% [150/542] 95% CI 24.0–31.6 vs 19.8% [101/510] 95% CI 16.5–23.4, Mann–Whitney *U* test *p* = 0.01). For the medium sized farms, the prevalence was 24.9% (123/494, 95% CI 21.2–28.9). The median number of cattle was 105 (range 1–935) in compliant and 84 (1–890) in non-compliant farms.

Non-compliances related to the adult cattle were more frequently detected in the small farms than in the large ones (22.6% [121/536] 95% CI 19.2–26.3 vs. 16.2% [80/495] 13.1–19.6, Mann–Whitney *U* test *p* = 0.03). The type of non-compliance was affected by the cattle herd size in farms with adult animals (Kruskal–Wallis *p* < 0.05 for all). The non-compliances related to ‘space’ and ‘management’ were more frequently detected in the small farms than in the medium and large ones (Table 5, Mann–Whitney *U* test *p* < 0.01 for both). The non-compliances related to ‘feeding and watering’ were more frequently detected in the large farms than in the medium sized farms (Table 5, *p* = 0.043). There was a significant difference in the non-compliance of herd size with the following two requirements (Kruskal–Wallis *p* < 0.001 for both): (1) ‘hooves are checked often enough and treated when necessary’ and (2) ‘there is enough clean water available for animals’. The first non-compliance occurred more often in the small farms than in the medium sized and large farms (7.6% [39/511] 95% CI 5.6–10.2 vs. 2.7% [13/480] CI 95% 1.5–4.4 and 2.9% [14/478] 95% CI 1.7–4.7, respectively, Mann–Whitney *U* test *p* < 0.01 for both), and the second non-compliance more often in the large farms than in the medium sized farms (4.7% [23/494] 95% CI 3.1–6.8 vs. 1.1% [5/476] 95% CI 0.4–2.2, *p* = 0.003).

In the calves, the prevalence of the non-compliances related to ‘space’ was affected by the herd size (Kruskal–Wallis *p* = 0.01): more non-compliances were detected in the small farms than in the large ones (16.5% [60/363] 95% CI 13.0–20.6 vs. 10.1% [49/485] 95% CI 7.7–13.0, Mann–Whitney *U* test *p* = 0.018). There was also a significant difference in the non-compliance of herd size with the following three requirements (Kruskal–Wallis *p* < 0.05 for all): (1) ‘calves are not tied up’ was more often detected as being non-compliant in the small farms than in the medium sized and large farms (5.4% [20/369] 95% CI 3.4–8.1 vs. 0.9% [4/466] 95% CI 0.3–2.0 and 0.2% [1/489] 95% CI 0.0–1.0, respectively, Mann–Whitney *U* test *p* < 0.001 for both); (2) ‘calves over 8 weeks of age are kept in a group box’ was more often detected as being non-compliant in the small and medium sized farms than in the large farms (11.2% [33/294] 95% CI 8.0–15.2 and 6.5% [27/412] 95% CI 4.5–9.3 vs. 1.6% [7/433] 95% CI 0.7–3.1, *p* < 0.001 for both); (3) ‘group boxes for calves are large enough’ was more often detected as being non-compliant in the medium sized farms than in the large ones (9.4% [38/404] 95% CI 6.8–12.5 vs. 5.0% [22/438] 95% CI 3.3–7.4, *p* = 0.042).

No statistically significant difference between the different sized pig farms in not complying with the welfare standards was found (Kruskal–Wallis *p* = 0.105). The percentage of small and large pig farms not complying with the welfare standards was 32.5% (38/117, 95% CI 24.5–41.3) and 31.9% (29/91, 95% CI 23.0–41.9), respectively, while the percentage was 22.1% (34/154, 95% CI 16.1–29.1) for the medium sized farms. There was no statistically significant difference between different sized pig farms in complying with the main welfare categories or individual requirements.

### 3.4. Effect of Production Type

There was no statistically significant difference in the overall proportion of non-compliant cattle farms with different production types (Kruskal–Wallis *p* = 0.14). However, in the adult cattle, there was a statistically significant difference between the farms with different production types in complying with the requirements related to ‘space’, ‘feeding and watering’ and ‘outdoor rearing and pasture’ (Kruskal–Wallis *p* < 0.01 for all). The non-compliances related to ‘space’ were more common in the farms with other cattle compared with the dairy cattle and suckler cow herds (Table 5, Mann–Whitney *U* test < 0.05 for both). The non-compliances related to ‘feeding and watering’ and ‘outdoor rearing and pasture’ were more common in suckler cow herds compared with the farms with other cattle and dairy cattle (Table 5, Mann–Whitney *U* test *p* < 0.05 for both). There was also a significant difference in farm type with the following requirements (Kruskal–Wallis *p* < 0.02 for all): (1) ‘each animal has a clean and adequate lying area’, (2) ‘feed and drinking water remain clean’, (3) ‘there is enough clean water available for animals’, (4) ‘ground of outdoor area remains properly dry’, and (5) ‘animals have adequate weather protection’. Excluding the requirement for adequacy of the lying area, which was most often detected as non-compliant in the farms with other cattle, the requirements were detected as non-compliant most often in suckler cow herds (Table 6).

In calves, there was a statistically significant difference between the different production types in complying with the space requirements (Kruskal–Wallis *p* < 0.01 for all): non-compliances in the farms with dairy cattle were detected more frequently than in the suckler cow herds (16.2% [144/890] 95% CI 13.9–18.7 vs. 6.9% [15/218] 95% CI 4.1–10.8, Mann–Whitney *p* = 0.005). There was also a significant difference in farm type with the following two requirements (Kruskal–Wallis *p* < 0.02 for both): (1) ‘calves over 8 weeks of age are kept in a group box’ and (2) ‘group boxes for calves are large enough’. These requirements were more often detected as non-compliant in dairy cattle (Table 6).

Of the inspected dairy cattle farms, 88.7% (833/939) kept their cattle tied for at least some part of a year, while the prevalence was 29.3% (77/186) and 45.1% (155/344) for the farms with suckler cows and for the farms categorized as ‘other’, respectively. The farms which kept cattle tied for at least part of the year were noticed to be more often linked to non-compliances compared with the farms which kept their cattle loose (27.2% [290/1065] 95% CI 24.6–30.0 vs. 17.5% [84/481] 95% CI 14.3–21.0, Mann–Whitney *U* test *p* < 0.001). The prevalence of the non-compliances concerning the calves was significantly higher if the farm kept the cattle tied than if they did not (19.6% [185/942] 95% CI 17.2–22.3 vs. 10.9% [44/403] 95% CI 8.2–14.2, Mann–Whitney *U* test *p* < 0.001). Further, if the farm kept the cattle tied, the calves more often had an inadequate lying area (6.3% [59/932] 95% CI 4.9–8.0 vs. 4.1% [16/390] 95% CI 2.5–6.4, *p* = 0.02), were kept too long in individual pens (7.7% [66/860] 95% CI 6.0–9.6 vs. 0.4% [1/279] 95% CI 0.0–1.7, Mann–Whitney *U* test *p* < 0.001) and the stocking density in a calf’s group box was too high (8.7% [73/841] 95% CI 6.9–10.7 vs. 3.5% [10/289] 95% CI 1.8–6.1, *p* = 0.007).

Of the farms with suckler cows, 58.6% (154/263) reared at least some part of their cattle outdoors year-round, while the prevalence was 10.0% (94/939) and 13.7% (47/344) for the farms with dairy cattle and farms categorized as ‘other’, respectively. The prevalence of the non-compliant cattle farms was clearly higher if the farm reared their cattle outdoors year-round than if they did not (29.5% [87/295] 95% CI 24.5–34.9 vs. 22.9% [287/1251] 95% CI 20.7–25.3, Mann–Whitney *U* test *p* = 0.018). In addition, the following requirements were more often detected as non-compliant in the farms rearing their adult cattle outdoors year-round than in the farms not rearing their cattle outdoors year-round (Table 7, Mann–Whitney *U* test *p* < 0.01 for all): (1) ‘cleanliness of facilities and equipment are adequately taken care of’, (2) ‘each animal has a clean and adequate lying area’, (3) ‘feed and drinking water remain clean’, and (4) ‘animals have adequate weather protection’. In calves, the following non-compliant requirements were more often detected in the farms rearing their adult cattle outdoors year-round than in the farms not rearing their cattle outdoors year-round (Table 7, Mann–Whitney *U* test *p* < 0.02 for all): (1) ‘facilities and equipment are safe for animals’, (2) ‘cleanliness of facilities and equipment are adequately taken care of’, (3) ‘each animal has a clean and adequate lying area’, and (4) ‘feed and drinking water remain clean’.

The prevalence of the non-compliant pig farms with farrow-to-finish units was 34.9% (59/169, 95% CI 28.0–42.3), while the prevalence was 22.6% (28/124, 95% CI 15.9–30.5) and 20.3% (14/69, 95% CI 12.1–30.9) for fattening and farrowing units, respectively (Kruskal–Wallis *p* = 0.020). The farrow-to-finish units had a statistically higher prevalence of non-compliances than the fattening and farrowing units (Mann–Whitney *U* test *p* < 0.05 for both). There was a statistically significant difference between the farms with different production types in complying with the requirements related to ‘management’ (Kruskal–Wallis *p* = 0.015): non-compliances were more frequently detected in the pig farms with a farrow-to-finish unit than in the farms with either a fattening or farrowing unit (26.6% [45/169] 95% CI 20.4–33.6 vs. 15.3% [19/124] 95% CI 9.8–22.4 and 13.0% [9/69] 95% CI 6.7–22.5, respectively, Mann–Whitney *U* test *p* < 0.05 for both).

The lack of enrichment material was clearly the most common individual non-compliance in all types of pig farm: overall 12.4% of the inspected farms did not have a sufficient amount of enrichment material and the farm types did not differ significantly (Kruskal–Wallis *p* = 0.33).

### 3.5. Regional Differences

The regions differed in the overall prevalence of the non-compliant cattle farms, ranging from 12.3% (9/73, 95% CI 6.3–21.3) to 33.1% (139/420, 95% CI 28.7–37.7) (Kruskal–Wallis *p* < 0.001). There was a significant difference in the regions with the following three requirements (Kruskal–Wallis *p* < 0.01 for all): (1) ‘each animal has a clean and adequate lying area’, (2) ‘hooves are checked often enough and treated when necessary’, and (3) ‘individual pens for calves are large enough’. Although the regions differed in their mean temperatures (1.9–6.2 °C, Kruskal–Wallis *p* = 0.038), the non-compliances did not correlate with the climate figures.

The prevalence of the non-compliant pig farms differed between the regions, ranging from 5.3% (1/19, 95% CI 0.6–22.1) to 37.8% (48/127, 95% CI 29.7–46.4) (Kruskal–Wallis *p* = 0.006).

### 3.6. Effect of the Inspection Season

The prevalence of the non-compliances in cattle farms during different seasons was 28.6% (34/119, 95% CI 21.0–37.1) in winter, 21.4% (46/215, 95% CI 16.3–27.2) in spring, 21.3% (133/625, 95% CI 18.2–24.6) in summer and 27.4% (161/587, 95% CI 23.9–31.1) in autumn. Although the non-compliances did not correlate with the climate figures in different regions, seasonal differences in the overall non-compliances were found (Kruskal–Wallis *p =* 0.038), the difference being statistically significant between the summer and autumn seasons (Mann–Whitney *U* test *p* = 0.041). Further, a higher number of the non-compliances in the adult cattle in the winter and autumn seasons than in spring was found (24.3% [28/115] 95% CI 17.2–32.8 and 22.1% [127/575] 95% CI 18.8–25.6 vs. 11.3% [24/213] 95% CI 7.5–16.0, respectively, Mann–Whitney *U* test *p* < 0.01 for both).

The prevalence of the non-compliances related to ‘feeding and watering’ and ‘outdoor rearing and pasture’ in the adult cattle differed depending on the season (Kruskal–Wallis *p* < 0.05 for both): the non-compliances related to ‘feeding and watering’ were detected more often in winter than in summer (Table 5, Mann–Whitney *U* test *p* = 0.02) and non-compliances related to ‘outdoor rearing and pasture’ more often in autumn than in spring (Table 5, *p* < 0.01). In the requirements for the adult cattle there was a statistically significant difference between the seasons as follows (Kruskal–Wallis *p* < 0.05 for all): the non-compliance of the requirement ‘there is enough clean water available for animals’ was more often recorded as non-compliant during the winter inspections than in the summer season (7.0% [8/114] 95% CI 3.4–12.8 vs. 1.1% [7/616] 95% CI 0.5–2.2, Mann–Whitney *U* test *p* < 0.01). During the autumn inspections, the requirements ‘each animal has a clean and adequate lying area’ and ‘animals have adequate weather protection’ were more often detected as non-compliant than in the summer season (11.5% [66/575] 95% CI 9.1–14.3 vs. 6.5% [39/604] 95% CI 4.7–8.6 and 4.3% [15/345] 95% CI 2.6–6.9 vs. 1.1% [5/439] 95% CI 0.4–2.5, respectively, Mann–Whitney *U* test *p* < 0.01 for both). Further, in autumn the requirement ‘ground of outdoor area remains properly dry’ was detected as non-compliant more often than in the spring season (6.7% [21/313] 95% CI 4.3–9.9 vs. 0.0% [0/95], Mann–Whitney *U* test *p* = 0.008). The prevalence of the farms with dairy cattle not complying with the requirement ‘tie-stalled dairy cows and heifers have access to pasture or exercise area outdoors’ was overall 4.4% (31/705, 95% CI 3.1–6.1) and in the inspections performed in the summer season the prevalence was 6.9% (21/304, 95% CI 4.5–10.2).

No seasonal variation in the inspection results in the calves and pig farms was detected (Kruskal–Wallis *p* > 0.05 for all).

## 4. Discussion

Based on the analysis of official EU animal welfare inspection reports on Finnish cattle and pig farms, it was discovered that approximately one quarter of the inspected farms do not comply with the minimum requirements imposed by the Finnish National Animal Welfare legislation. The prevalence of non-compliant farms in this study is quite small considering that most of the inspected farms (75%) had previous problems with welfare or marking and registering. It appears that the situation on farms can change. The fact that the data was biased while consisting of two kinds of farms (75% sampled based on risk and 25% by random sampling) puts some limitations on the study. The data may not present an average Finnish farm. However, risk factors that may predict non-compliances in cattle and pig farms were detected. The non-compliances of the cattle farms were more often related to housing conditions, while in the pig farms they were related more often to management. The reasons for this might be that the influence of the pig industry on pig farming is stronger and thus pig houses are more uniform and standardized compared with cattle houses, which are more variable with different housing types. Secondly, most of the pig farms belong to the Finnish healthcare system, mandating a regular farm visit of a healthcare veterinary surgeon, while only approximately 60% of the cattle farms belong to the system [13].

We found more frequent prevalence of non-compliances in cattle farms with small herd size and tie-stall housing. Tie-stall housing is recognized as a risk factor for non-compliance in dairy cattle in Sweden [7]. The finding is probably explained by older premises with restricted space and facilities as nearly all new cattle houses in Finland are free stalls, and these stalls have a higher number of animals compared with tie-stalls [14]. In addition, the more animals in a farm, the more likely is the regularity of visits by a veterinary surgeon. For example, if a dairy farm is receiving veterinary medicines for future use, a regular healthcare veterinary surgeon visit is required. Thus, a veterinary surgeon visits a farm with 60 dairy cows four times a year but once every second week for farms with more than 300 dairy cows [15]. 

It was found that small tie-stall dairy barns have, in particular, problems with calf housing conditions, i.e., overage calves were kept in single pens, calves were tied, or calves were group housed with a too high stocking density. This was the case despite national programmes aiming to improve calf housing conditions launched by the Finnish Food Safety Authority Evira and other interest groups in the early 2010s. Within the framework of the programmes, educative information was disseminated, and all cattle farmers were provided written advice about the protection of calves [16,17]. However, in the light of the results of this study, the results of these efforts have not been satisfactory. The requirements for housing are clearly stated in the Finnish national legislation thus the non-compliances cannot be explained by difficulty in interpreting the legislation. Farmer incompetence to find legislative information, reluctance to comply due to investment costs, or inability to see the future benefits of taking good care of calf well-being may be reasons behind the non-compliances. Emphasis should be given to measures to improve calf housing conditions as the most common non-compliances directly affect calf welfare. For example, a lack of sufficient space decreases calf playing behavior [18], which is important for the welfare of young animals [19].

There is no official information about the number of Finnish farms rearing cattle outdoors year-round, but based on our data, almost every fifth farm inspected reared at least some part of their cattle outdoors. Outdoor rearing has several benefits for cattle welfare, such as comfortable lying space [20], less lameness [21] and better hoof [22] and udder health [23], however, it was shown that this husbandry is associated with higher risk of non-compliances. In Finland, the wide temperature changes between the four seasons pose challenges to outdoor rearing. In particular, a lack of sufficient shelter, an inadequate lying area and dirtiness were common problems in the farms rearing their cattle outdoors year-round. According to Finnish legislation, cattle reared outdoors must have adequate weather protection and a bedding area inside [24]. The weather protection is crucial for cattle not only during cold winters [25,26] but also in other seasons including summer when shelter is used to protect the animals from heat stress [27,28,29,30]. As it is shown here, nearly every tenth Finnish farm rearing cattle outdoors did not have adequate weather protection. As a comparison, in France only 2.3% of the farms rearing their cattle outdoors did not have adequate protection against adverse weather and predators [8]. The farmers rearing their cattle outdoors in Finland should be familiar with the challenges related to Nordic conditions. This type of husbandry provides, when well-managed, many welfare benefits.

It was evident that certain non-compliances were seasonally variable in cattle farms but not in the pig farms. The plausible explanation for this isthat in the Finnish conditions pig houses are more closed and are not influenced by cold weather conditions as strongly as cattle houses are. Thus, the seasons during which cattle farms had more difficulties to fulfil all the animal welfare requirements were also the seasons when the weather in Finland is the most challenging: cold winter and rainy autumn seasons. Lundmark Hedman et al. [7] have found that there is a higher probability of non-compliance in Swedish cattle farms during the winter season. The extreme winter temperatures (below −30 °C) freezes the water supply systems unless well insulated and this probably explains some non-compliance issues in winter. Further, autumn is rainy and outdoor moisture is high thus imposing challenges to the bedding area. Cattle prefer a dry lying surface, and they spend much more time standing when only wet bedding is available [31]. In addition to directly affecting the welfare of cattle, a low hygiene level of the lying surface and inadequate bedding were recognized as risk factors for the two main welfare problems of dairy cattle: mastitis and lameness [32]. It is essential for farmers to understand the importance of a well-managed lying area for the welfare and health of cattle.

It was also found that in the summer season, keeping tie-stalled dairy cows inside without access to the outdoors was a common problem. Due to the practice of restriction of movement of tie-stalled dairy cows and heifers, Finnish animal welfare legislation demands 60-day access to pasture or an alternative exercise area outdoors during the summer to ensure that cattle can behave naturally: moving, social behavior and foraging. There are many health benefits for cattle having access to pasture, for example less lameness [22] and mastitis [23], and welfare benefits, for example the possibility to graze, which is a natural behavior of cattle [33].

No clear explanation for the higher prevalence of non-compliances in pig farms with farrow-to-finish units than with fattening and farrowing units was found. The most common non-compliances were lack of enrichment material and record-keeping. It is possible that pig farms should concentrate more on a specific farming type to be successful, but it should, however, be noted that the farrow-to-finish units have many welfare and biosecurity advantages: the risk of diseases is smaller when the production chain is closed and there is less transportation of pigs. Further investigations are warranted to explain the issues of lack of enrichment and record-keeping in farrow-to-finish farms, which otherwise seems easier to comply with. This might be due to the lack of information and motivation or the fear of blocking of the manure removal system, among the farmers. The absence of records may indicate more severe non-compliance [34]. The lack of enrichment material was the most common problem in pig farm inspections and this non-compliance has a direct effect on the welfare of pigs because without enrichment material pigs may not satisfy their natural exploratory behavior [34]. Furthermore, when they can fulfil this need, the risk of a range of adverse welfare consequences lessens [35,36]. The directive 2008/120/EC concerning pig welfare [37] requires that pigs ‘must have permanent access to a sufficient quantity of material to enable proper investigation and manipulation activities’. In Finland, Evira [38] has instructed that if this is not possible, for example because of the risk of blocking the manure removal system, pigs need to have continuous access to toys, such as balls, rods or chains, which should be varied, and pigs should be offered straw, hay, newspapers, etc., at least twice a day which they can root around. Although it is easy to inspect whether pigs have toys or not, it is not possible to inspect whether the toys are varied, and the adequate assessment of other enrichment material is even more difficult.

Some farmers in Finland still cut or grind the piglets’ teeth routinely to prevent injuries to other piglets and the sow’s udder caused by piglets fighting with their littermates to establish a teat order. The practice causes pain to piglets and carries a risk of infection [39,40], and it is thus regulated by legislation. The practice is only allowed when done on less than 7-day-old piglets when a sow has teat injuries. Before performing the procedure, the housing conditions should be checked and improved [37]. In order to decrease the need for the practice, farmers should be encouraged to try other ways to diminish fighting, for example by using more enrichment material [41] or not cross-fostering with pigs older than 24 hours [42]. 

As has been shown in this study, non-compliances were related to different farm types. Thus, efficient dissemination of specific information targeting specific farmer might raise better awareness than blanket instructions to all farmers. The inspections should be based on risk assessments and the season is to be taken into consideration when scheduling risk-based farm inspections, ensuring efficient and timely inspections. In addition, thematic inspections, i.e., related to specific non-compliance risks such as dairy cattle summer pasture access, housing conditions of calves in small tie-stall dairy farms, or use of enrichment material in pig farms, should be performed. The data obtained from inspections should also be used for developing inspection instructions for animal welfare authorities. A degree of difference in the occurrence of non-compliances in different regions was found, and it is possible that the differences are due to the different practical implementation by inspectors. Most of the differences were related to the requirements, which are not very detailed or specific, for example adequate lying area and hoof care when necessary, and thus leave room for interpretation. The present instructions for the authorities concerning these requirements should be reassessed and complemented as it is essential that the farmers are treated equally. Unequal treatment has been found to cause anxiety in farmers [43,44]. However, differences related to the requirements which are unequivocal were also detected, i.e., size of calf single pens. The information on whether the inspection was pre-informed or not is not included in the inspection report, thus it is not known how well the Finnish inspectors obey the EC Regulation No 882/2004 [4] prohibiting of pre-informing about the inspections. This also has influence on the evaluation and comparison of the inspection results, as pre-informing is likely to affect the inspection result [3,7]. 

Although the evaluation of inspection reports itself was not part of this study, the report system based on checklists is not very descriptive for the farmer or other readers, as the scale is very narrow, i.e., either compliant or not. Thus, the present inspection result does not describe anything about the severity, length or extent of the non-compliance. To increase the information content, the scale could be expanded, the requirements categorized into severity classes or a total estimation could be given. In France, the farms are graded into four classes based on the inspection: they can be either fully compliant or slightly, moderately or severely non-compliant [8]. A four-grade smiley-face system is already used in the Finnish food inspection system, and similar grading in animal welfare inspection system could be useful not only to farmers to help them better understand the severity of problems but also to other authorities, such as police or prosecutors involved in the legal process. The implementation of animal welfare legislation could be better targeted in farms which are known to be severely non-compliant.

## 5. Conclusions

The official inspection reports contain valuable information about the welfare problems in farms. The reports not only expose the problems related to basic animal husbandry which may have a significant impact on the welfare of animals but also reveal farm characteristics such as farm size or production type which may be associated with a higher risk. The data obtained from inspections should be better utilized not only in risk analysis but also in targeting farmer education, improving the instructions for authorities, and in making the inspections more uniform.

## Figures and Tables

**Table 1:** Prevalence of cattle farms not complying with the main welfare categories or the checklist requirements concerning adult cattle.

Required Compliance	Non-Compliant Farms *n* ^a^, % ^b^ (95% CI)
**General requirements for premises**	103, **6.9** (5.7–8.2)
Facilities and equipment are	
safe for animals	46, **3.1** (2.3–4.0)
easily cleanable	16, **1.1** (0.6–1.7)
suitable for disinfection	15, **1.0** (0.6–1.6)
Cleanliness of facilities and equipment are adequately taken care of	73, **4.9** (3.9–6.1)
Floors do not cause damage to animals	26, **1.8** (1.2–2.5)
Liquid secretions are properly removed or absorbed into bedding	52, **3.4** (2.2–4.8)
Pest control is taken care of	9, **0.6** (0.3–1.1)
Animals are easily removed from shelter	5, **0.3** (0.1-0.7)
**Space requirements**	144, **9.7** (8.3–11.3)
Each animal has a clean and adequate lying area	128, **8.5** (7.2–10.0)
Stalls are appropriate (1065)	22, **2.1** (1.3–3.3)
Equipment for stalls is appropriate	6, **0.6** (0.3–1.2)
**Environment**	34, **2.3** (1.7–3.2)
Temperature is good for animals	3, **0.2** (0.1–0.5)
Lighting is suitable for animals and adequate for proper inspection and care of animals	22, **1.5** (1.0–2.2)
Air quality and moisture are good for animals	12, **0.9** (0.4–1.4)
In case of mechanical ventilation, the system is checked daily and there is an alarm system which is also tested regularly	4, **1.4** (0.5–3.3)
The noise is quiet enough	2, **0.1** (0.0–0.4)
**Management**	108, **7.1** (5.9–8.5)
There are enough staff to take care of the animals	1, **0.1** (0.0–0.3)
Hooves are checked often enough and treated when necessary	66, **4.5** (3.5–5.6)
Social hierarchy is considered	6, **0.4** (0.2–0.8)
There are no electronic stalls and tails are not kept tied all the time	2, **0.2** (0.0–0.5)
There is proper care for diseased and injured animals	18, **1.4** (0.8–2.1)
Sick and injured animals are placed, where appropriate, in separate compartments	12, **0.9** (0.5–1.6)
Record of medical treatment is kept	24, **1.7** (0.8–2.1)
Record of dead animals is kept	8, **0.5** (0.3–1.0)
Killing animals is done appropriately	6, **0.4** (0.2–0.8)
**Feeding and watering**	67, **4.4** (3.4–5.5)
Animals have enough adequate feed	17, **1.1** (0.6–1.7)
Feeding systems are available for all animals	4, **0.6** (0.2–1.5)
In case of a mechanical feeding system, the system is checked daily and there is a back-up system	2, **0.5** (0.1–1.7)
Feed and drinking water remain clean	26, **1.7** (1.2–2.5)
There is enough clean water available for animals	39, **2.6** (1.9–3.5)
In case of a mechanical drinking system, the system is checked daily and there is a back-up system	5, **0.4** (0.1–0.8)
In case of an automatic drinking or feeding system, animals have become accustomed to it	0, **0.0**
**Outdoor rearing and pasture**	96, **8.5** (7.0–10.2)
Tie-stalled dairy cows and heifers have access to pasture or exercise area outdoors	34, **4.2** (3.0–5.7)
Ground of outdoor area remains properly dry	35, **4.1** (2.9–5.5)
Animals (excluding animals reared outside year-round) have adequate weather protection	24, **2.4** (1.6–3.5)
Fences are suitable, safe and in good condition	11, **1.2** (0.7–2.1)
There are appropriate facilities for isolating and treating animals	7, **1.0** (0.4–1.9)
Animals outside year-round have adequate weather protection	24, **8.1** (5.4–11.7)

^a^ The number of farms which did not comply with the requirement; ^b^ The prevalence of farms which did not comply with the requirement; it was counted from farms in which the requirement was relevant. The bold is to emphasize the mean percentages.

**Table 2:** Prevalence of cattle farms not complying with the main welfare categories or the checklist requirements concerning calves.

Required Compliance	Non-Compliant Farms *n* ^a^, % ^b^ (95% CI)
**General requirements for premises**	64, **4.8** (3.8–6.1)
Facilities and equipment are	
safe for animals	24, **1.8** (1.2–2.7)
easily cleanable	13, **1.0** (0.6–1.6)
suitable for disinfection	13, **1.0** (0.6–1.6)
Cleanliness and disinfection of facilities and equipment are adequately taken care of	47, **3.6** (2.7–4.7)
Floors do not cause damage to animals	8, **0.6** (0.3–1.2)
Liquid secretions are properly removed or absorbed into bedding	32, **2.4** (1.2–4.1)
Pest control is taken care of	7, **0.5** (0.2–1.0)
Animals are easily removed from shelter	0, **0.0**
**Space requirements**	183, **13.9** (12.1–15.8)
Calves are not tied up	25, **1.9** (1.3–2.7)
Each animal has a clean and suitable lying area	75, **5.7** (4.5–7.0)
Calves less than 2 weeks of age have a well-littered lying area	27, **3.1** (2.1–4.4)
Individual pens for calves are large enough	24, **3.2** (2.1–4.7)
There is a medical reason if calves are kept in a closed-wall pen	7, **1.0** (0.4–1.3)
Calves over 8 weeks of age are kept in a group box	67, **5.9** (4.6–7.4)
There is a medical reason if calves are kept alone	1, **0.9** (0.1–1.1)
Group boxes for calves are large enough	83, **7.3** (5.9–9.0)
**Environment**	23, **1.8** (1.1–2.6)
Temperature is good for animals	2, **0.2** (0.0-0.5)
Lighting is suitable for animals and adequate for proper inspection and care of animals	13, **1.0** (0.6–1.7)
Air quality and moisture are good for animals	6, **0.5** (0.2–1.0)
In case of mechanical ventilation, the system is checked daily and there is an alarm system which is also tested regularly	5, **1.9** (0.7–4.2)
The noise is quiet enough	0, **0.0**
**Management**	36, **2.7** (1.9–3.6)
There are enough staff to take care of the animals	13, **1.0** (0.6–1.7)
Calves are checked at least twice daily	3, **0.2** (0.1–0.6)
There is proper care for diseased and injured animals	9, **0.8** (0.4–1.4)
Sick and injured animals are placed, where appropriate, in separate compartments	6, **0.5** (0.2–1.1)
Record of medical treatment is kept	12, **0.9** (0.5–1.6)
Record of dead animals is kept	5, **0.4** (0.1–1.0)
Killing animals is done appropriately	6, **0.5** (0.2–1.0)
Dehorning is done when calves are under 4 weeks	1, **0.2** (0.0–0.8)
**Feeding and watering**	43, **3.2** (2.4–4.2)
Animals have enough adequate feed	6, **0.4** (0.2–0.9)
Calves are fed at least twice a day	3, **0.2** (0.1–0.6)
In case of a mechanical feeding system, the system is checked daily and there is a back-up system	0, **0.0**
Feed and drinking water remain clean	18, **1.3** (0.8–2.1)
There is enough pure water available for animals	11, **0.8** (0.4–1.4)
Calves are given something to drink at least twice dailyIn hot weather there is clean water always available for calves	3, **0.2** (0.1–0.6)
In case of sick calves there is water available all the time	7, **0.8** (0.4–1.6)
There are enough water places for animals	9, **0.7** (0.4–1.3)
In case of a mechanical drinking system, the system is checked daily and there is a back-up system	11, **1.0** (0.5–1.8)
In case of an automatic drinking or feeding system, animals have become accustomed to it	1, **0.1** (0.0–0.5)

^a^ The number of farms which did not comply with the requirement; ^b^ The prevalence of farms which did not comply with the requirement; it was counted from farms in which the requirement was relevant. The bold is to emphasize the mean percentages.

**Table 3:** Prevalence of pig farms not complying with the main welfare categories or the checklist requirements.

Required Compliance	Non-Compliant Farms *n* ^a^, % ^b^ (95% CI)
**General requirements for premises**	26, **7.2** (4.9–10.2)
Facilities and equipment are	
safe for animals	7, **1.9** (0.9–3.8)
easily cleanable	1, **0.3** (0.0–1.3)
suitable for disinfection	1, **0.3** (0.0–1.3)
Cleanliness and disinfection of facilities and equipment are adequately taken care of	9, **2.5** (1.2–4.5)
Floors do not cause damage to animals	5, **1.4** (0.5–3.0)
Liquid secretions are properly removed or absorbed into bedding	11, **3.0** (1.6–5.2)
Pest control is taken care of	6, **1.7** (0.7–3.4)
There is equipment for fire and rescue	2, **0.6** (0.1–1.8)
**Space requirements**	29, **8.0** (5.5–11.1)
Each animal has a clean and suitable lying area	11, **3.0** (1.6–5.2)
Pigs kept in groups have enough space	14, **4.0** (2.3–6.4)
There is enough space for boar	7, **3.7** (1.7–7.1)
There is enough space behind sow in farrowing crate	0, **0.0**
Piglets have a dry and appropriate lying area where they can lie down at the same time	0, **0.0**
In case of free-farrowing, piglets are protected	0, **0.0**
**Environment**	12, **3.3** (1.8–5.6)
Temperature is good for animals	0, **0.0**
Lighting is suitable for animals and adequate for proper inspection and care of animals	8, **2.2** (1.1–4.1)
Air quality and moisture are good for animals	0, **0.0**
In case of mechanical ventilation, the system is checked daily and there is an alarm system which is also tested regularly	4, **3.6** (1.2–8.4)
The noise is quiet enough	0, **0.0**
**Management**	12, **20.2** (16.3–24.5)
There are enough staff to take care of the animals	1, **0.3** (0.0–1.3)
Pigs are checked at least daily	0, **0.0**
Pigs can see other pigs	0, **0.0**
Pigs have straw or other suitable enrichment material	45, **12.4** (9.3–16.1)
Sows are given suitable material for construction of the farrowing nest	4, **1.8** (0.6–4.2)
Mixing groups is avoided	2, **0.6** (0.1–1.8)
In case of fighting, appropriate measures have been taken	0, **0.0**
Record of medical treatment is kept	17, **4.8** (2.9–7.4)
Record of dead animals is kept	10, **2.8** (1.4–4.9)
Killing animals is done appropriately	3, **0.8** (0.2–2.2)
Piglets have appropriate warmer when needed	0, **0.0**
Sows are treated against parasites	1, **0.4** (0.0–2.0)
Teeth of the piglets are cut or ground only if there are injuries to sow nipples and it is done on under 8-day-old piglets	8, **4.4** (2.1–8.2)
Piglets are castrated before the age of 8 days	7, **3.1** (1.4–6.0)
Piglets are weaned over 4 weeks of age	1, **0.4** (0.0-2.0)
Tails are not cut	0, **0.0**
**Feeding and watering**	20, **5.5** (3.5–8.2)
Animals have enough adequate feed	0, **0.0**
Pigs are fed at least daily	0, **0.0**
In case of a mechanical feeding system, the system is checked daily and there is a back-up system	0, **0.0**
Pigs kept in groups can eat at the same time unless feed is not ad libitum	10, **3.1** (1.6–5.5)
Feed and drinking water remain clean	3, **0.8** (0.2–2.2)
In case of a mechanical drinking system, the system is checked daily and there is a back-up system	0, **0.0**
There is water available all the time after age 2 weeks	10, **2.8** (1.4–4.9)
In case of an automatic drinking or feeding system, animals have become accustomed to it	0, **0.0**

^a^ The number of farms which did not comply with the requirement; ^b^ The prevalence of farms which did not comply with the requirement; it was counted from farms in which the requirement was relevant. The bold is to emphasize the mean percentages.

**Table 4:** Descriptive information for inspected farms and inspection season divided by farm type.

	Cattle Farms % (*n*)	Pig Farms % (*n*)
Small (<50 cattle or 250 pigs)	35.1 (542)	32.3 (117)
Medium (50–100 cattle or 250–750 pigs)	32.0 (494)	42.5 (154)
Large (>100 cattle or 750 pigs)	33.0 (510)	25.1 (91)
Dairy cattle	60.7 (939)	NA
Suckler cow herd	17.0 (263)	NA
Other cattle	22.3 (344)	NA
Farrow-to-finish unit	NA	46.7 (169)
Farrowing unit	NA	19.1 (69)
Fattening unit	NA	34.3 (124)
Southern Finland	17.0 (263)	16.0 (58)
Southwestern Finland	8.2 (127)	35.6 (129)
Western and Inland Finland	27.2 (420)	35.1 (127)
Eastern Finland	24.8 (383)	7.7 (28)
Northern Finland	18.1 (280)	5.2 (19)
Lapland	4.7 (73)	0.3 (1)
Winter	7.7 (119)	11.9 (43)
Spring	13.9 (215)	12.7 (46)
Summer	40.4 (625)	33.1 (120)
Autumn	38.0 (587)	42.3 (153)

Total number of reports on cattle farms = 1546; total number of reports on pig farms = 362; NA = Not Applicable.

**Table 5:** Percentage of non-compliant cattle farms in different main welfare categories concerning adult cattle, divided by herd size, production type and inspection season.

	Non-Compliant Farms % (95% CI)					
Variable (N ^a^)	General Requirements for Premises	Space Requirements	Environment	Management	Feeding and Watering	Outdoor Rearing and Pasture
**Herd Size**						
Small (404–536)	**8.4** (6.2–11.0)	**12.8** (10.1–15.8)	**3.6** (2.3–5.5)	**10.4** (8.1–13.2)	**3.8** (2.4–5.6)	**9.7** (7.1–12.8)
medium (405–489)	**7.0** (5.0–9.6)	**8.6** (6.3–11.3)	**1.9** (0.9–3.4)	**5.1** (3.4–7.3)	**3.1** (1.8–4.9)	**8.4** (6.0–11.4)
large (323–495)	**5.1** (3.4–7.3)	**7.5** (5.4–10.1)	**1.4** (0.6–2.8)	**5.5** (3.7–7.7)	**6.3** (4.4–8.7)	**7.1** (4.7–10.3)
*p*-value ^b^	NS	0.01	NS	0.001	0.04	NS
**Production Type**						
dairy cattle (751–939)	**5.9** (4.5–7.5)	**8.1** (6.5–10.0)	**2.7** (1.8–3.8)	**6.8** (5.3–8.6)	**3.1** (2.1–4.3)	**6.3** (4.7–8.2)
suckler cow herd (201–263)	**8.8** (5.7–12.7)	**9.0** (5.9–13.0)	**0.8** (0.2–2.6)	**6.5** (4.0–9.9)	**7.3** (4.6–10.9)	**14.2** (10.2–19.1)
other cattle (142–318)	**8.4** (5.7–11.8)	**15.1** (11.4–19.4)	**2.6** (1.2–4.8)	**8.5** (5.8–11.9)	**5.7** (3.5–8.6)	**10.6** (6.3–16.4)
*p*-value ^c^	NS	0.002	NS	NS	0.006	<0.001
**Inspection Season**						
winter (72–115)	**4.5** (1.7–9.5)	**12.5** (7.3–19.6)	**1.8** (0.4–5.7)	**10.4** (5.8–17.0)	**9.6** (5.2–15.9)	**5.6** (1.9–12.7)
spring (147–213)	**4.2** (2.1–7.6)	**6.7** (3.9–10.7)	**1.4** (0.4–3.7)	**5.2** (2.8–8.8)	**4.2** (2.1–7.6)	**2.0** (0.6–5.3)
summer (499–617)	**6.9** (5.1–9.2)	**7.8** (5.9–10.2)	**1.7** (0.9–2.9)	7.2 (5.9–10.1)	**3.3** (2.1–4.9)	**8.2** (6.0–10.9)
autumn (414–587)	**8.2** (6.2–10.7)	**12.2** (9.7–15.1)	**3.5** (2.2–5.3)	**6.4** (4.6–8.7)	**4.5** (3.1–6.5)	**11.6** (8.8–14.9)
*p*-value ^d^	NS	0.02	NS	NS	0.03	0.003

^a^ The number of farms in which the category was relevant; ^b^ Kruskal–Wallis *T* test between the groups with different herd size; ^c^ Kruskal–Wallis *T* test between the groups with different production type; ^d^ Kruskal–Wallis *T* test between the farms inspected in different seasons; NS = Not Statistically Significant; *p* > 0.05. The bold is to emphasize the mean percentages.

**Table 6:** Pairwise comparison of different cattle production types not complying with certain individual requirements.

	Requirement	Dairy Cattle	Suckler Cow Herd	Other Cattle
		% (*n*/N) 95% CI	% (*n*/N) 95% CI	% (*n*/N) 95% CI
Adult cattle	Each animal has a clean and adequate lying area	**7.2** (67/935) 5.6–9.0 ^b^	NS	**12.4** (39/314) 9.1–16.4 ^a^
	Feed and drinking water remain clean	**0.9** (8/938) 0.4–1.6 ^b^	**5.0** (13/261) 2.8–8.1 ^a^	**1.6** (5/316) 0.6–3.4 ^b^
	There is enough clean water available for animals	**1.6** (15/938) 0.9–2.6 ^b^	**4.9** (13/263) 2.8–8.1 ^a^	NS
	Ground of outdoor area remains properly dry	**1.8** (10/553) 0.9–3.2 ^b^	**9.7** (5/100) 1.9–10.6 ^a^	NS
	Animals have adequate weather protection	**0.9** (6/632) 0.4–1.9 ^b^	**6.1** (13/213) 3.5–9.9 ^a^	NS
Calves	Calves over 8 weeks of age are kept in a group box	**7.2** (61/842) 5.6–9.1 ^a^	**1.0** (1/103) 0.1–4.4 ^b^	**2.6** (5/194) 1.0–5.6 ^b^
	Group boxes for calves are large enough	**8.6** (71/821) 6.9–10.7 ^a^	**0.9** (1/110) 0.1–4.1 ^b^	NS

^a^ Statistically significantly (Mann–Whitney *U* test *p* < 0.05) higher than ^b^; NS = difference from other production types not statistically significant. The bold is to emphasize the mean percentages.

**Table 7:** Statistically significant difference for certain requirements between the cattle farms rearing their cattle outdoors year-round versus farms which do not rear outdoors year-round.

	Requirement	Outdoor Rearing Year-Round % (*n*/N) 95% CI	Not Year-Round Outdoor Rearing % (*n*/N) 95% CI	*p*-Value ^a^
Adult cattle	Cleanliness of facilities and equipment are adequately taken care of	**2.2** (6/277) 0.9–4.4	**0.8** (10/1212) 0.4–1.5	<0.001
	Each animal has a clean and adequate lying area	**13.1** (38/289) 9.6–17.4	**7.4** (90/1215) 6.0–9.0	0.002
	Feed and drinking water remain clean	**5.8** (17/295) 3.5–8.9	**0.7** (9/1221) 0.4–1.3	<0.001
	Animals have adequate weather protection	**7.1** (19/256) 4.5–10.7	**0.6** (4/692) 0.2–1.4	<0.001
Calves	Facilities and equipment are safe for animals	**5.4** (13/239) 3.1–8.9	**1.0** (11/1074) 0.5–1.8	0.004
	Cleanliness of facilities and equipment are adequately taken care of	**1.7** (4/237) 0.6–4.0	**0.8** (9/1072) 0.4–1.5	<0.001
	Each animal has a clean and adequate lying area	**8.9** (22/247) 5.8–12.9	**4.9** (53/1075) 3.8–6.3	0.015
	Feed and drinking water remain clean	**3.1** (8/259) 1.5–5.7	**0.9** (10/1082) 0.5–1.6	0.007

^a^ Mann–Whitney *U* test. The bold is to emphasize the mean percentages.
